# Comorbid Psychiatric Factors Contributing to Adolescent Alcohol and Other Drug Use

**Published:** 2002

**Authors:** Deborah Deas, Suzanne Thomas

**Affiliations:** Deborah Deas, M.D., M.P.H., is an associate professor and Suzanne Thomas, Ph.D., is an assistant professor in the Institute of Psychiatry’s Center for Drug and Alcohol Programs at the Medical University of South Carolina in Charleston, South Carolina

**Keywords:** comorbidity, adolescent, AODU (alcohol and other drug use), behavioral and mental disorder, psychological AODU disorder theory, peer relations, environmental factors, family AODU history, risk factors, emotional depression, anxiety, conduct disorder, attention deficit disorder with hyperactivity, genetic linkage, literature review

## Abstract

Alcohol and other drug (AOD) use is common among adolescents, and its consequences pose important public health problems. Consequently, it is essential to understand the numerous factors that place adolescents at risk for AOD use. These factors include psychological and psychiatric influences (e.g., comorbid psychiatric disorders) as well as peer, environmental, and family factors. The impact of these factors may be moderated by other influences, such as the adolescents’ previous AOD use experience and gender. A thorough understanding of the factors that influence adolescent AOD use, and how their effects may change as adolescents age, is critical for the development of effective prevention and treatment efforts.

Alcohol and other drug (AOD) use among adolescents is a major public health problem and has been linked to such adverse consequences as car crashes, suicide, delinquency, criminal behaviors, and psychological difficulties. Furthermore, the presence of AOD use problems during adolescence is the single most predictive factor for adult AOD dependence (Swadi 1999). Therefore, an understanding of the factors that place adolescents at risk for AOD use and abuse is critical for the development of effective prevention efforts. Many variables influence adolescents’ risk of AOD use, including psychological factors; psychiatric factors, such as coexisting psychiatric disorders; and peer, environmental, and family variables. Some of these influences are more powerful than others; furthermore, their relative impact may change over the course of adolescence. This article discusses the various risk factors and the roles they play in shaping adolescents’ AOD use throughout adolescence, focusing specifically on psychiatric risk factors. For an in-depth discussion of risk factors in general, see Hawkins and colleagues (1992).

## Psychological Factors

In general, the term “psychological factors” refers to patterns of thought and behavior that exist along a continuum in the general population—including, for example, personality traits, self-esteem, and coping skills. It is a somewhat all-inclusive term that is used here to refer to all nonpathological factors related to thought patterns and behaviors that may impact an adolescent’s risk of developing AOD use problems.

Several studies have identified one psychological factor that is consistently related to an increased risk of AOD use problems in both adolescents and adults—a personality pattern of high novelty seeking, low harm avoidance, and high reward dependence. This constellation of traits was first implicated in alcoholism risk in adults, but has also been shown to apply to adolescents at risk for AOD use (for a review see Swadi 1999).

Another psychological factor that has been shown to be predictive of both the initiation and continuation of AOD use is high aggressivity (Swadi 1999). For example, Reinherz and colleagues (2000) found that both teacher-rated and self-rated aggressivity at age 9 was predictive of AOD use disorders at age 21.

Low self-esteem also has been implicated in many disorders in adolescents; however, surprisingly little evidence suggests that it is a risk factor for adolescent AOD abuse (Swadi 1999). Thus, although adolescents with AOD use disorders may experience low self-esteem, it is unlikely that this low self-esteem was a causal factor for the development of the disorder.

Stressful or traumatic life events also increase adolescents’ risk for developing use problems. For example, Kilpatrick and colleagues (2000) found that adolescents who witnessed or experienced physical and/or sexual assault were at greater risk for developing AOD use disorders than were adolescents without such experiences. Similarly, in an analysis of factors related to initiation and increase of AOD use, Wills and colleagues (2001) showed that the number of stressful life events experienced by adolescents was related to both the initiation and continuation of AOD use.

## Psychiatric Factors

Psychiatric factors differ from psychological factors in that they represent emotional and behavioral conditions of a severity that warrant classification as mental disorders, which meet specific diagnostic criteria as stipulated in the *Diagnostic and Statistical Manual on Mental Disorders, Fourth Edition* (DSM–IV) (American Psychiatric Association 1994). These psychiatric disorders can co-occur with AOD use disorders, and the presence of psychiatric disorders can serve as a risk factor for the development of AOD use disorders in both adolescents and adults. Although the term “comorbidity” in general refers to the co-occurrence of two psychiatric disorders, in the AOD abuse field it typically refers to the co-occurrence of an affective or anxiety disorder with an AOD use disorder.

In discussing comorbidity as a risk factor for AOD use disorders, it is important to note the order of onset of the two disorders (i.e., whether they start simultaneously or whether one predates the other). Obviously, for a psychiatric disorder to be a risk factor for an AOD use disorder, its onset must precede that of the AOD use problem.^1^ To determine the order of onset of two disorders, researchers ideally use prospective, longitudinal studies, in which healthy subjects are followed over long periods of time and monitored for the development of the disorders under investigation. Although this approach is preferable to retrospective studies in which subjects with the disorders estimate the age at which they began experiencing a particular problem, prospective studies are difficult and costly to conduct and therefore used less commonly. Instead, retrospective studies and cross-sectional studies (which include participants of different ages to estimate the probable order of onset) are the most common study designs and provide most of the information in the field of comorbidity research.

Even if the onset of an affective or anxiety disorder predates the AOD use disorder, it is possible that a third factor predisposes a person to both disorders. In these cases, the AOD use may not necessarily be in response to the preceding psychiatric disorder; rather, both disorders develop independently in response to the predisposing factor. In short, onset of a psychiatric disorder prior to an AOD use disorder is a necessary but not sufficient condition to conclude that the psychiatric disorder is a risk factor for the AOD use disorder. Although the self-medication hypothesis—which proposes that a person begins to use AODs to alleviate the symptoms of a psychiatric disorder—makes intuitive sense, it is not necessarily an accurate depiction of the situation.

The following sections discuss the co-occurrence of some psychiatric disorders with AOD use disorders and their potential role as risk factors for the development of AOD use disorders. In reviewing the issue of comorbidity, it is important to note the population on which each study is based. That is, comorbidity rates vary with the study population (e.g., general population versus treatment sample), and results based on one type of sample may not generalize to other samples.

### Depression

The seriousness of depression in adolescents and the necessity of its assessment are well recognized by clinicians and researchers. Depression during adolescence is closely linked to suicidal thoughts and behaviors, especially in the adolescents who also use AODs. For example, Wagner and colleagues (1996) found that depression and AOD use commonly coexisted among juniors and seniors in high school who attempted suicide.

Several studies have reported an association between depression and AOD use in adolescents. In a study of inpatient adolescents, 73 percent of adolescents who used AODs met diagnostic criteria for depression. Furthermore, in 80 percent of those cases, the depressive symptoms predated the AOD use (Deas-Nesmith et al. 1998*a*). This temporal relationship also has been observed by other investigators (Burke et al. 1994; Rohde et al. 1991), although other studies have not confirmed it (Hovens et al. 1994). Nevertheless, these observations suggest that depression may be a risk factor for AOD use and AOD use disorders in adolescents.

### Anxiety Disorders

Similar to depression, anxiety disorders have also been found to predate AOD use, and adolescents with anxiety disorders may self-medicate their symptoms by using AODs. Female adolescents may be especially prone to self-medication of anxiety symptoms. For example, Rohde and colleagues (1996) reported that alcohol use among female high school students was associated with anxiety disorders that preceded the alcohol problems.

Although few studies have examined the association between AOD use and specific anxiety disorders in adolescents, the existing literature suggests that two anxiety disorders in particular—social anxiety disorder and post-traumatic stress disorder (PTSD)—may be related to the risk of AOD use disorders in adolescents.

Social anxiety disorder, which refers to an intense fear and avoidance of social scrutiny, has been particularly associated with AOD abuse, and especially alcohol use, in adults (Kushner et al. 1990). Deas-Nesmith and colleagues (1998*b*) explored the prevalence of comorbid psychiatric disorders and AOD use disorders among adolescents who were seeking treatment for AOD use problems. The investigators found that social anxiety disorder was the most common co-occurring psychiatric disorder among adolescents with an AOD use disorder— 60 percent of the AOD-abusing adolescents met the criteria for social anxiety disorder. The investigators also found that when an anxiety disorder coexisted with AOD use, the anxiety symptoms generally predated AOD use by about two years, suggesting that the anxiety disorder could be a risk factor for AOD use. Because the sample studied consisted solely of adolescents seeking treatment for AOD use disorders, however, these results may not apply to non-treatment-seeking or community samples.

The relationship of PTSD to AOD use also has been studied mostly in adult samples, although Kilpatrick and colleagues (2000) recently explored PTSD as a risk factor for AOD use problems in adolescents. These investigators found that PTSD increased the risk of abuse of several illicit drugs. One theory regarding the role of PTSD and traumatic life events as risk factors for AOD use disorders in adolescents posits that the affected adolescents often use AODs to cope with the traumatic experiences. This theory is supported by a study exploring the traumatic histories of adolescents with alcohol use disorders (Clark et al. 1997*a*). This study found that the alcohol-abusing adolescents were 6 to 12 times more likely to have a history of physical abuse and 18 to 21 times more likely to have a history of sexual abuse compared with control adolescents. Furthermore, the study suggested that the relationship of PTSD to AOD abuse in adolescents may be gender specific because the association of PTSD and alcohol dependence was stronger in females than in males (Clark et al. 1997*b*).

### Conduct Disorder

Conduct disorder, commonly referred to as childhood antisocial behavior, has been shown to be particularly strongly associated with the onset as well as continuation of AOD use. For example, the presence of conduct disorder and related disorders predicts early initiation of regular alcohol use in early adolescence, onset of alcohol-related problems in adolescence, and development of alcohol use disorders. The relationship of conduct disorder to AOD use is fully described by Clark and colleagues, pp. 109–115, of this journal issue.

### Attention Deficit Hyperactivity Disorder

Attention deficit hyperactivity disorder (ADHD), which is characterized by the constellation of impulsivity, hyperactivity, and inattention, commonly coexists with AOD use among adults and adolescents. There has been much discussion and debate whether people with ADHD are more likely to abuse AODs than people without the disorder. Studies suggest that the relationship between ADHD and AOD use in adolescents is moderated by other factors. For example, Biederman and colleagues (1997) prospectively followed adolescents with and without ADHD for four years and found no differences in the rates of AOD use between the two groups. Instead, the presence of conduct disorder and bipolar disorder was a more valid predictor of AOD use than ADHD status. Other studies conducted on adults and adolescents indicate that a person’s level of cognitive functioning moderates the relationship between ADHD and AOD use. That is, people with lower cognitive functioning are more likely to use AODs in response to their hyperactivity than are people with higher cognitive functioning, possibly because the latter employ more effective coping techniques (Span and Earleywine 1999; Dawes et al. 2000).

## Other Risk Factors for Adolescent AOD Use and Abuse

In addition to psychological factors and psychiatric comorbidity, numerous other factors can influence adolescents’ AOD use behaviors and can increase the risk of initiating and escalating AOD use. These influences include peer factors, environmental factors, and family factors.

### Peer Factors

One of the changes that characterize adolescence is the development of an increasing independence. Part of this process involves looking outside the family for role models. Consequently, although parental guidance and approval remain important influences on adolescent behavior, peer guidance and approval become increasingly powerful and valued. These shifts in influences shape numerous aspects of adolescent behavior, including AOD use.

A large body of literature has addressed specific aspects of these shifting influences as they relate to AOD use (for a review, see Swadi 1999). These studies consistently demonstrate that the single most important factor related to an adolescent’s AOD use is whether his or her friends use AODs. For example, in a study of 1,700 adolescents assessed yearly from the 7th to the 9th grade, good correlations (as indicated by correlation coefficients of 0.53–0.56) existed between the level of AOD use in the respondent and the number of peers who used AODs (Wills et al. 2001). In fact, the relationship between these two variables was stronger than with any other factor measured (e.g., number of negative life events and coping style indices). It is also important to note that the association between peer use and adolescent self-use is typically strongest in young adolescents (i.e., less than 16 years of age). Moreover, peer use is most strongly related to variables related to the *initiation* of AOD use rather than to variables related to *continuation* of use (Swadi 1999).

### Environmental Factors

In the literature regarding environmental risk factors for adolescent AOD use, the term “environmental” is typically used in one of two ways. In one usage, “environmental” refers to the physical environment and broader social conditions that surround the adolescent (e.g., socioeconomic status [SES] and living in a crime-ridden area). In this usage, “environmental factors” typically means influences that are separate from family factors or peer influences. In the other, more general usage, the term “environmental” refers to any influence that is not genetically determined and therefore includes peer factors or non-genetic family factors.

Studies analyzing environmental factors according to the first definition of the term report varying results. Nevertheless, these studies identified the following factors as particularly important:

- *Homelessness*. In a study involving a large sample of homeless youth, 71 percent of the respondents had an AOD use disorder (Kipke et al. 1997). The study did not determine, however, whether AOD use problems resulted in homelessness or stemmed from it.
- *Low SES.* A low SES may also be a risk factor for adolescent AOD use. However, it probably acts through indirect mechanisms, such as a greater incidence of stressful life events and limited access to a college education and the opportunity to “mature out” of AOD use habits (Reinherz et al. 2000; Dawes et al. 2000; Schulenberg et al. 1996).
- *Living in an urban environment.* A study conducted in Finland (which may not generalize to other populations) found that living in an urban environment was related to a decrease in the likelihood of abstinence in adolescents, as probably AODs are more easily available in urban areas than in rural areas (Rose 1998). However, no evidence suggests that urban-dwelling adolescents are inherently at greater risk to develop an AOD use *problem* than are rural-dwelling adolescents.

Several studies have investigated the role of environmental factors according to the second, more general definition of the term. These studies have yielded consistent results regarding the relative strength of environmental (i.e., non-genetic) influences on adolescent AOD use. The data show that the strength of environmental influences decreases from mid- to late adolescence (i.e., between 16 and 18 years of age), whereas the strength of genetic influences increases (Rose 1998).

Although these studies were not intended to investigate the reason for this effect, it is tempting to speculate about underlying causes. One hypothesis is that the motives for AOD use, or at least alcohol use, change dramatically during adolescence. For example, an adolescent’s initial motives for drinking may be to fit in socially or to satisfy a curiosity. Once the young person starts drinking, however, his or her continued alcohol use may be driven by the physical and psychological effects of alcohol consumption. Substantial research has shown that a person’s response to alcohol is genetically influenced (these genetic influences are discussed in more detail in the following section). Therefore, as a person’s motives for drinking change and the response to alcohol becomes increasingly important in determining drinking levels, it is likely that environmental influences lose their impact and genetic predisposition becomes more important.

### Family Factors

Although the quest for independence drives adolescents to look outside the family for guidance regarding AOD use, as evidenced by the increasing importance of peer influences, family factors continue to influence adolescent AOD use. Family variables continue to exert a strong influence not only because most adolescents still value their family members as models of behavior, but also because these factors encompass such a wide range of influences. For example, family factors include the following:

- Family structure (i.e., the size of the family and whether the child lives with the mother, father, or both parents)
- Family dynamics (i.e., parenting style and sibling relationships)
- AOD use by the parents and/or siblings
- Genetic influences (i.e., the presence or absence of a family history of AOD use).

Family factors can protect against adolescent AOD use and abuse (for a review, see Vakalahi 2001), or they can place an individual at risk. Regarding the latter, several studies report that adolescents whose parents or siblings have past or current AOD use problems are themselves at an increased risk of developing AOD use disorders (Reinherz et al. 2000; Kilpatrick et al. 2000). This association could be related either to a shared genetic predisposition or to nongenetic factors, such as impaired parenting skills, greater availability of AODs, and/or greater perceived parental acceptance of AOD use. Interestingly, a study found that even if a father is abusing AODs, his presence in the home may provide greater protection against the development of adolescent AOD use problems than his absence (Tarter et al. 2001). In other words, adolescents who live only with their mothers are more likely to develop AOD use problems than adolescents living with both parents, even if the father abuses AODs.

Larger family size has also been shown to increase the risk of adolescent AOD use disorders (Reinherz et al. 2000). In addition, these investigators found that for males only, being born to younger parents (i.e., less than 21 years old at the time of the child’s birth) increased the risk of AOD use problems nearly sixfold. Finally, the parental influence against AOD use is tempered by whether the adolescent is involved in a peer group that supports AOD use (Gerrard et al. 1999).

The study of genetic influences on AOD use in adolescence is still in its infancy, but it appears certain that genetic factors increase in strength as an adolescent ages (Rose 1998). As suggested earlier, this finding may be related to the adolescent’s experience with AOD use. This experience typically increases with age, and as it increases, the adolescent’s reasons for continued use may change, and the level of response to the drug, which is possibly inherited, may determine whether AOD use continues.

This hypothesis has been especially well studied regarding the physiological response to alcohol. Several investigators have shown that college-age sons of alcoholics show a lower subjective response to the sedative effects of alcohol (delivered in a controlled setting) compared with college students without a family history of alcoholism (for a review, see Pollock 1992). This effect has also been shown in daughters of alcoholics (Schuckit et al. 2000). Other investigators have shown that people with a family history of alcoholism are more sensitive to the stimulating effects of alcohol (Newlin and Thomson 1990). Whether people experience less sedation or more stimulation with alcohol, both scenarios suggest that an inherited differential response to alcohol may make alcohol more reinforcing for people with a genetic predisposition to alcoholism. This theory may apply especially to people who drink to feel alcohol’s effects (e.g., alcohol-experienced, older adolescents) rather than to people who drink to fit in or experiment with alcohol (e.g., alcohol-inexperienced, younger adolescents) and may help explain why genetic influences gain strength and environmental influences lose strength as an adolescent ages.

Indirect support for this hypothesis also comes from the results of the first genetic study on drug use conducted exclusively on adolescents. In that study— which did not address alcohol use— McGue and colleagues (2000) found that a significant genetic influence existed on the use and abuse of nicotine but not on the use and abuse of illicit substances (i.e., marijuana, opiates, PCP, cocaine, sedatives, inhalants, and amphetamines). This finding supports the notion that genetic factors play a role in the continued use of a drug. Nicotine often is the first drug used by adolescents, and therefore is the drug with which most adolescents have “experience.” Whether a nicotine-experienced adolescent continues to smoke therefore may be influenced by the effects he or she obtains from nicotine, which could be genetically influenced. In contrast, with illicit drugs, adolescents typically are inexperienced in their use, so use of these drugs may more likely be driven by social and/or environmental factors rather than genetic factors.

## Conclusions

Most people will experiment with AODs for the first time during adolescence, and some people will continue to use AODs throughout adolescence and into adulthood. Researchers and clinicians now have a better understanding of the factors that increase the risk of AOD use in adolescence and contribute to its continuation and escalation. Adolescence is a dynamic period characterized by changes in many realms (e.g., physical, emotional, hormonal, and psychological), including AOD use experience. Therefore, it is important that this period be viewed not as a single “snapshot” of development, but rather as a period when risk factors for AOD use can and will change in their relative impact over time. For example, the literature suggests that peer influences and environmental influences are especially important in early adolescence and in the initiation of AOD use. Genetic influences become more powerful for the continuation of AOD use as the adolescent enters adulthood; family, psychiatric, and psychological influences appear to be stable, constant influences. These shifts in the relevance of various risk factors must be considered in the development of appropriate prevention and intervention measures targeted at adolescents.

## Footnotes

1 Theoretically, there could be exceptions to this rule where a “latent” psychiatric disorder may contribute to AOD use, which, in turn, results in the psychiatric disorder’s full manifestation and diagnosis.
